# Supplementary material for: Structure and stability of the designer protein WRAP-T and its permutants
Source: Sci Rep. 2021 Sep 22;11:18867. doi: 10.1038/s41598-021-98391-0 (PMC8458387; doi:10.1038/s41598-021-98391-0)
Supplement: Supplementary file 1 — Supplementary Information. [file 41598_2021_98391_MOESM1_ESM.pdf]

Table S1: Amino acid and DNA sequences

Protein	Amino Acid sequence	DNA sequence
**nvWRAP-T**	MGHSSSVTGVAFSPDGQTI<br>ASASDDKTVKLWNRNGQL<br>LQTLTGHSSSVTGVAFSPD<br>GQTIASASDDKTVKLWNRN<br>GQLLQTLTGHSSSVTGVAF<br>SPDGQTIASASDDKTVKLW<br>NRNGQLLQTLTGHSSSVTG<br>VAFSPDGQTIASASDDKTV<br>KLWNRNGQLLQTLTGHSS<br>SVTGVAFSPDGQTIASASD<br>DKTVKLWNRNGQLLQTLTG<br>HSSSVTGVAFSPDGQTIAS<br>ASDDKTVKLWNRNGQLLQ<br>TLTGHSSSVTGVAFSPDGQ<br>TIASASDDKTVKLWNRNGQ<br>LLQTLT	ATGGGTCACAGTTCTAGTGTTACCGGAGT<br>AGCTTTTTCGCCCGATGGACAGACCATT<br>GCCTCTGCTTCTGACGATAAGACTGTAAA<br>GTTATGGAATCGCAATGGCCAACTGCTTC<br>AAACCTTAACGGGCCACAGTAGCAGCGT<br>CACGGGGGTTGCTTTCAGTCCGGATGGA<br>CAAACTATCGCTTCTGCCAGCGACGACA<br>AAACTGTCAAACTTTGGAATCGTAACGGC<br>CAGTTATTACAAACTCTGACGGGGCATTC<br>AAGTTCCGTTACAGGTGTCGCATTTTCAC<br>CGGATGGCCAGACGATTGCCAGCGCGTC<br>AGATGATAAGACTGTCAAGTTATGGAACC<br>GGAACGGTCAACTTCTGCAAACACTTAC<br>CGGGCATTCGTCGAGTGTCACCGGTGTA<br>GCCTTCTCTCCGGATGGCCAGACCATTG<br>CATCAGCTTCCGATGATAAGACTGTAAAG<br>CTTTGGAACCGGAACGGGCAATTATTACA<br>GACGTTGACTGGCCATTCGTCCTCAGTC<br>ACGGGTGTGGCATTCTCTCCTGATGGTC<br>AAACAATTGCTTCCGCAAGTGACGATAAA<br>ACTGTTAAGTTGTGGAACCGTAATGGACA<br>GTTATTACAAACACTGACCGGACATTCAA<br>GCTCAGTGACTGGAGTCGCTTTTAGCCC<br>GGATGGGCAAACTATCGCTTCAGCCTCT<br>GACGATAAGACCGTCAAGCTTTGGAACA<br>GAAACGGACAATTACTGCAGACGTTAACT<br>GGTCATTCGTCTAGCGTAACAGGAGTCG<br>CTTTTAGTCCAGACGGCCAAACGATTGC<br>CTCCGCGTCCGATGATAAAACCGTCAAG<br>CTTTGGAATCGGAATGGGCAGTTGCTTC<br>AGACTCTGACATAA
**v31WRAP-T**	QTIASASDDKTVKLWNRNG<br>QLLQTLTGHSSSVTGVAFS<br>PDGQTIASASDDKTVKLWN<br>RNGQLLQTLTGHSSSVTGV<br>AFSPDGQTIASASDDKTVK<br>LWNRNGQLLQTLTGHSSS<br>VTGVAFSPDGQTIASASDD<br>KTVKLWNRNGQLLQTLTGH<br>SSSVTGVAFSPDGQTIASA<br>SDDKTVKLWNRNGQLLQT<br>LTGHSSSVTGVAFSPDGQT<br>IASASDDKTVKLWNRNGQL<br>LQTLTGHSSSVTGVAFSPD<br>GQTIASASDDKTVKLWNRN<br>GQLLQTLTGHSSSVTGVAF<br>SPDG	CAAACGATTGCCTCAGCCAGTGACGATA<br>AAACTGTCAAGTTATGGAATCGTAATGGC<br>CAACTGCTGCAGACACTGACAGGACACT<br>CTTCCAGCGTAACTGGCGTGGCTTTTTC<br>GCCTGACGGTCAAACAATTGCGTCAGCC<br>TCTGATGATAAGACAGTAAAACTGTGGAA<br>CCGCAACGGACAATTGCTTCAGACCTTG<br>ACCGGACATTCCTCGTCAGTCACGGGTG<br>TTGCGTTTAGTCCCGATGGGCAAACTATT<br>GCGTCGGCCTCCGACGACAAGACCGTC<br>AAGTTATGGAATCGCAATGGTCAATTGCT<br>GCAAACGTTAACGGGACACAGTTCCTCT<br>GTTACCGGAGTAGCCTTTTCCCCTGACG<br>GACAAACCATTGCCTCCGCCAGTGACGA<br>TAAAACAGTCAAGCTTTGGAATCGTAATG<br>GGCAATTACTGCAAACGCTTACGGGTCAT<br>TCAAGTTCGGTTACTGGTGTAGCGTTTAG<br>CCCAGACGGGCAAACCATCGCTTCTGCC<br>TCGGATGACAAAACGGTTAAGCTGTGGA<br>ACCGCAATGGTCAGTTGTTACAAACATTG<br>ACAGGTCACAGTTCGTCTGTGACAGGGG<br>TCGCTTTTTCACCCGATGGCCAAACAATT<br>GCTTCTGCAAGCGATGACAAAACTGTGA<br>AGTTATGGAACCGTAACGGGCAGTTGCT<br>TCAGACTTTAACTGGGCACAGTAGCTCTG<br>TCACGGGGGTAGCATTCAGCCCCGATGG<br>TCAGACTATCGCGTCCGCGTCAGATGAC<br>AAAACGGTGAAGTTGTGGAACCGTAACG<br>GCCAATTATTGCAAACTCTTACAGGGCAC<br>TCTTCATCCGTTACTGGGGTTGCGTTTTC<br>ACCTGACGGATAA

**v22WRAP-T**	MDKTVKLWNRNGQLLQTL<br>TGHSSSVTGVAFSPDGQTI<br>ASASDDKTVKLWNRNGQL<br>LQTLTGHSSSVTGVAFSPD<br>GQTIASASDDKTVKLWNRN<br>GQLLQTLTGHSSSVTGVAF<br>SPDGQTIASASDDKTVKLW<br>NRNGQLLQTLTGHSSSVTG<br>VAFSPDGQTIASASDDKTV<br>KLWNRNGQLLQTLTGHSS<br>SVTGVAFSPDGQTIASASD<br>DKTVKLWNRNGQLLQTLTG<br>HSSSVTGVAFSPDGQTIAS<br>ASDDKTVKLWNRNGQLLQ<br>TLTGHSSSVTGVAFSPDGQ<br>TIASASD	ATGGACAAGACTGTAAAGCTTTGGAACC<br>GCAATGGCCAATTACTGCAGACGCTGAC<br>GGGTCACTCGTCTAGCGTAACTGGAGTA<br>GCGTTTTCACCAGACGGCCAGACCATCG<br>CATCAGCCTCTGACGATAAAACCGTAAAA<br>TTATGGAATCGGAACGGCCAGCTGCTGC<br>AAACTCTTACAGGCCACTCTTCCTCGGTG<br>ACTGGAGTGGCATTTTCCCCCGACGGGC<br>AGACAATAGCTAGCGCCTCTGATGACAAG<br>ACTGTTAAACTGTGGAACAGAAATGGTCA<br>ACTTTTACAAACACTTACCGGACATTCGA<br>GCTCCGTTACTGGCGTAGCGTTCTCCCC<br>AGACGGGCAAACGATTGCATCTGCATCA<br>GATGATAAAACAGTCAAGCTGTGGAACC<br>GGAATGGTCAACTGCTGCAGACGCTTAC<br>AGGTCATTCATCTAGCGTAACCGGTGTTG<br>CGTTTAGCCCTGACGGTCAAACTATAGCC<br>TCTGCATCTGATGACAAGACAGTCAAATT<br>GTGGAATCGCAACGGGCAGCTGCTTCAG<br>ACCCTTACTGGACATTCTTCAAGTGTTAC<br>CGGAGTTGCCTTCAGTCCTGATGGACAG<br>ACTATTGCTTCTGCTTCTGACGATAAAACT<br>GTGAAGCTGTGGAATCGGAACGGACAGC<br>TGTTGCAGACACTGACAGGACACAGCTC<br>GTCCGTTACAGGCGTTGCTTTCTCACCT<br>GACGGCCAGACGATTGCTAGTGCAAGTG<br>ACGACAAGACGGTCAAACTTTGGAATCG<br>TAACGGGCAGCTTCTTCAGACATTAACCG<br>GCCACTCTAGTTCAGTTACCGGGGTCGC<br>TTTTTCTCCAGATGGTCAAACCATTGCTA<br>GTGCCTCGGACTAA
**v13WRAP-T**	MGQLLQTLTGHSSSVTGVA<br>FSPDGQTIASASDDKTVKL<br>WNRNGQLLQTLTGHSSSV<br>TGVAFSPDGQTIASASDDK<br>TVKLWNRNGQLLQTLTGHS<br>SSVTGVAFSPDGQTIASAS<br>DDKTVKLWNRNGQLLQTLT<br>GHSSSVTGVAFSPDGQTIA<br>SASDDKTVKLWNRNGQLL<br>QTLTGHSSSVTGVAFSPDG<br>QTIASASDDKTVKLWNRNG<br>QLLQTLTGHSSSVTGVAFS<br>PDGQTIASASDDKTVKLWN<br>RNGQLLQTLTGHSSSVTGV<br>AFSPDGQTIASASDDKTVK<br>LWNRN	ATGGGACAGTTATTGCAAACCTTGACTGG<br>CCACAGCAGCTCAGTAACCGGAGTCGCG<br>TTCTCACCGGACGGTCAAACAATAGCGT<br>CAGCATCTGACGACAAGACTGTGAAACT<br>TTGGAACAGAAATGGTCAACTTTTACAGA<br>CGCTGACTGGCCATTCCTCTTCAGTCACT<br>GGCGTAGCCTTCAGTCCAGATGGTCAAA<br>CCATTGCTTCAGCTAGCGATGACAAGAC<br>CGTTAAGTTATGGAACAGAAATGGACAAC<br>TGCTGCAAACTCTGACTGGTCACAGCTC<br>GTCTGTCACTGGAGTCGCCTTTTCGCCA<br>GACGGTCAGACGATTGCCTCAGCTTCCG<br>ACGATAAAACCGTTAAATTGTGGAATCGG<br>AACGGACAATTACTGCAGACTTTAACAGG<br>TCACTCGTCCTCGGTAACGGGAGTTGCC<br>TTCTCTCCAGACGGTCAGACTATAGCAAG<br>TGCATCCGACGATAAGACCGTAAAGTTGT<br>GGAACCGGAACGGTCAACTGCTTCAAAC<br>CTTAACAGGGCACTCTTCCTCTGTTACTG<br>GAGTGGCCTTTAGTCCAGATGGCCAAAC<br>GATAGCGTCGGCTTCGGATGACAAAACA<br>GTGAAACTGTGGAACCGGAACGGCCAGT<br>TGCTTCAGACATTGACCGGCCATAGTAGT<br>TCGGTTACGGGTGTTGCTTTCAGTCCTG<br>ATGGGCAAACTATCGCTTCCGCCTCAGA<br>CGACAAGACGGTGAAGCTTTGGAACCGG<br>AACGGACAATTACTTCAGACCCTGACAG<br>GCCACAGTAGCAGCGTTACGGGCGTTGC<br>TTTCAGTCCGGACGGGCAAACGATAGCT<br>TCGGCAAGTGATGATAAAACGGTAAAGCT<br>TTGGAACCGCAACTAA

Table S2: Crystallization conditions

Name	Protein Concentration (mg/ml)	Reservoir solution
nvWRAP-T	10	0.1M Citric acid pH 4.0, 10%(w/v)PEG 6000
v31WRAP-T	10	0.2M Ammonium formate 20% (w/v) PEG 3350
v22WRAP-T	10	0.2M Sodium thiocyanate pH 6.9, 20%(w/v)PEG 3350
v13WRAP-T	10	1.0 M Lithium chloride, 0.1 M HEPES pH7.0, 30% (w/v) PEG 6000

Table S3: Data-collection and refinement statistics of X-ray structures. Values in parentheses are for the outer shell.

	**nvWRAP-T**	**v31WRAP-T**	**v22WRAP-T**	**v13WRAP-T**
**PDB entry**	7BIE	7BID	7BIF	7BIG
**Data collection**				
Diffraction source	IO3, DLS	IO4, DLS	IO3, DLS	IO3, DLS
Wavelength	0.9763	0.9763	0.9763	0.9763
Resolution range	59.76-1.80 (1.84-1.80)	44.64-1.80 (1.84-1.80)	57.37-1.40 (1.42-1.40)	52.13-1.80 (1.84-1.80)
Space Group	$C2$	$P2_12_12_1$	$P2_1$	$P2_12_12_1$
a,b,c	79.66, 48.497, 121.95	44.64, 61.40, 90.41	63.85, 72.89, 93.55	64.93, 81.48, 87.42
α,β,γ	90, 101.47, 90	90, 90, 90	90, 96.01, 90	90, 90, 90
Reflections (measured/unique)	276861/41838	222824/26008	1073302/166377	567324/43750
Completeness	98.6 (97.5)	99.8 (97.3)	99.2 (86.5)	100 (100)
Mean I/sigma	11.9 (5.5)	55.4 (21.6)	25.5 (6.2)	15.4 (2.5)
Multiplicity	6.6 (6.2)	8.6 (5.0)	6.5 (4.9)	13.0 (12.2)
Rpim	0.039 (0.114)	0.009 (0.019)	0.016 (0.094)	0.028 (0.281)
CC(1/2)	0.993 (0.959)	1.000 (0.998)	1.000 (0.969)	0.997 (0.915)
Wilson B factor	16.7	11.32	8.8	20.2
**Refinement statistics**				
R factor/Rfree	0.173/0.216	0.1476/0.1827	0.156/0.0.181	0.195/0.243
No. atoms in structure	4679	2479	9822	4520
Protein	4153	2153	8461	4129
Ligand	13	0	0	0
Water	513	326	1361	391
**R.m.s deviation**				
Bond lengths	0.007	0.016	0.006	0.004
Bond angles	0.995	1.34	1.010	0.727
Chiral volume	0.0692	0.101	0.101	0.054
**Ramachandran plot**				
Favorable	95.25	95.42	94.98	95.77
Allowed	4.75	4.58	5.02	4.23
**Average B factor**	18.0	14.0	13.0	27.0

Table S4: Amino acid and DNA sequences of failed designs

Protein	Amino Acid sequence	DNA sequence
**Based on 1vyh**	FQTKESIRTLNGHRHSVTSI<br>SFHPNGPFIVSGSADKTIKV<br>WDFQTKESIRTLNGHRHSV<br>TSISFHPNGPFIVSGSADKTI<br>KVWDFQTKESIRTLNGHRH<br>SVTSISFHPNGPFIVSGSAD<br>KTIKVWDFQTKESIRTLNGH<br>RHSVTSISFHPNGPFIVSGS<br>ADKTIKVWDFQTKESIRTLN<br>GHRHSVTSISFHPNGPFIVS<br>GSADKTIKVWDFQTKESIRT<br>LNGHRHSVTSISFHPNGPFI<br>VSGSADKTIKVWDFQTKESI<br>RTLNGHRHSVTSISFHPNG<br>PFIVSGSADKTIKVWD	TTCCAGACTAAGGAGTCCATCCGTACCC<br>TTAATGGTCATCGCCATTCGGTAACCAG<br>CATTAGCTTCCACCCTAATGGTCCCTTCA<br>TTGTCTCTGGCAGTGCAGATAAAACTAT<br>CAAGGTATGGGACTTCCAAACAAAAGAG<br>TCCATTCGCACTCTTAACGGACACCGCC<br>ATTCGGTAACTTCCATCTCATTCCATCCA<br>AACGGTCCGTTCATCGTGAGTGGCTCA<br>GCCGATAAAACGATCAAGGTGTGGGATT<br>TTCAAACCAAAGAATCGATACGCACTCT<br>GAACGGGCATCGTCACTCGGTAACCTC<br>CATCTCATTCCATCCCAACGGTCCCTTTA<br>TCGTGTCTGGGAGTGCAGACAAAACGA<br>TCAAAGTATGGGATTTCCAGACTAAGGA<br>GTCCATCCGTACCCTTAATGGTCATCGC<br>CATTCGGTAACCAGCATTAGCTTCCACC<br>CTAATGGTCCCTTCATTGTCTCTGGCAG<br>TGCAGATAAAACTATCAAGGTATGGGACT<br>TCCAAACAAAAGAGTCCATTCGCACTCT<br>TAACGGACACCGCCATTCGGTAACTTCC<br>ATCTCATTCCATCCAAACGGTCCGTTCAT<br>CGTGAGTGGCTCAGCCGATAAAACGATC<br>AAGGTGTGGGATTTTCAAACCAAAGAAT<br>CGATACGCACTCTGAACGGGCATCGTCA<br>CTCGGTAACCTCCATCTCATTCCATCCC<br>AACGGTCCCTTTATCGTGTCTGGGAGTG<br>CAGACAAAACGATCAAAGTATGGGATTT<br>TCAAACCAAGGAAAGTATCCGCACATTG<br>AACGGCCACCGCCACTCGGTGACTTCA<br>ATCAGTTTCCATCCGAATGGGCCATTTAT<br>CGTATCTGGGTCCGCGGATAAGACAATC<br>AAAGTGTGGGAC
**Based on 4mk0**	IQGQQTRTLTGHNGAITSLS<br>FAPDGRLFVTGSDDATLKL<br>WDLRAIQGQQTRTLTGHNG<br>AITSLSFAPDGRLFVTGSDD<br>ATLKLWDLRAIQGQQTRTLT<br>GHNGAITSLSFAPDGRLFVT<br>GSDDATLKLWDLRAIQGQQ<br>TRTLTGHNGAITSLSFAPDG<br>RLFVTGSDDATLKLWDLRAI<br>QGQQTRTLTGHNGAITSLS<br>FAPDGRLFVTGSDDATLKL<br>WDLRAIQGQQTRTLTGHNG<br>AITSLSFAPDGRLFVTGSDD<br>ATLKLWDLRAIQGQQTRTLT<br>GHNGAITSLSFAPDGRLFVT<br>GSDDATLKLWDLRA	ATTCAGGGGCAGCAAACACGTACCCTG<br>ACGGGGCATAACGGGGCCATTACTAGTC<br>TGTCCTTTGCACCGGATGGCCGTTTGTT<br>CGTGACTGGAAGTGACGATGCCACGCT<br>GAAATTGTGGGATTTACGCGCTATCCAA<br>GGTCAACAAACTCGTACACTTACTGGAC<br>ACAACGGTGCCATCACTTCACTTTCATT<br>CGCACCCGACGGGCGTTTATTTGTTACT<br>GGGTCAGATGATGCTACCTTGAAACTTT<br>GGGATTTGCGTGCTATCCAAGGCCAACA<br>GACGCGCACCCTGACAGGTCATAACGG<br>AGCGATCACTTCGTTGTCGTTCGCCCCT<br>GACGGACGCCTGTTCGTGACCGGTAGC<br>GACGACGCGACTCTTAAACTGTGGGATC<br>TTCGCGCCATCCAAGGGCAACAAACCC<br>GCACTCTGACTGGCCATAATGGCGCGAT<br>CACCAGTCTTTCCTTCGCGCCAGATGGT<br>CGTCTTTTTGTGACTGGATCCGACGACG<br>CTACATTAAAACTTTGGGATTTGCGTGC<br>GATCCAAGGGCAACAGACCCGTACTCTT<br>ACTGGGCACAACGGTGCCATTACGAGTT<br>TGAGTTTTGCTCCTGACGGTCGCTTGTT<br>CGTCACAGGGTCCGATGATGCAACTTTG<br>AAATTATGGGACTTGCGCGCTATTCAGG<br>GCCAACAAACTCGTACGTTGACGGGAC<br>ACAACGGTGCCATCACGTCCCTTTCGTT<br>CGCTCCTGACGGACGTTTGTTTGTTACG<br>GGGTCCGACGACGCCACCTTGAAGCTG<br>TGGGATTTACGTGCGATCCAAGGCCAGC<br>AAACTCGTACATTGACAGGACATAATGG<br>GGCGATTACATCGCTGTCTTTTGCTCCG<br>GACGGCCGCCTGTTCGTAACAGGCTCA<br>GATGATGCGACATTGAAACTTTGGGACT<br>TACGCGCT

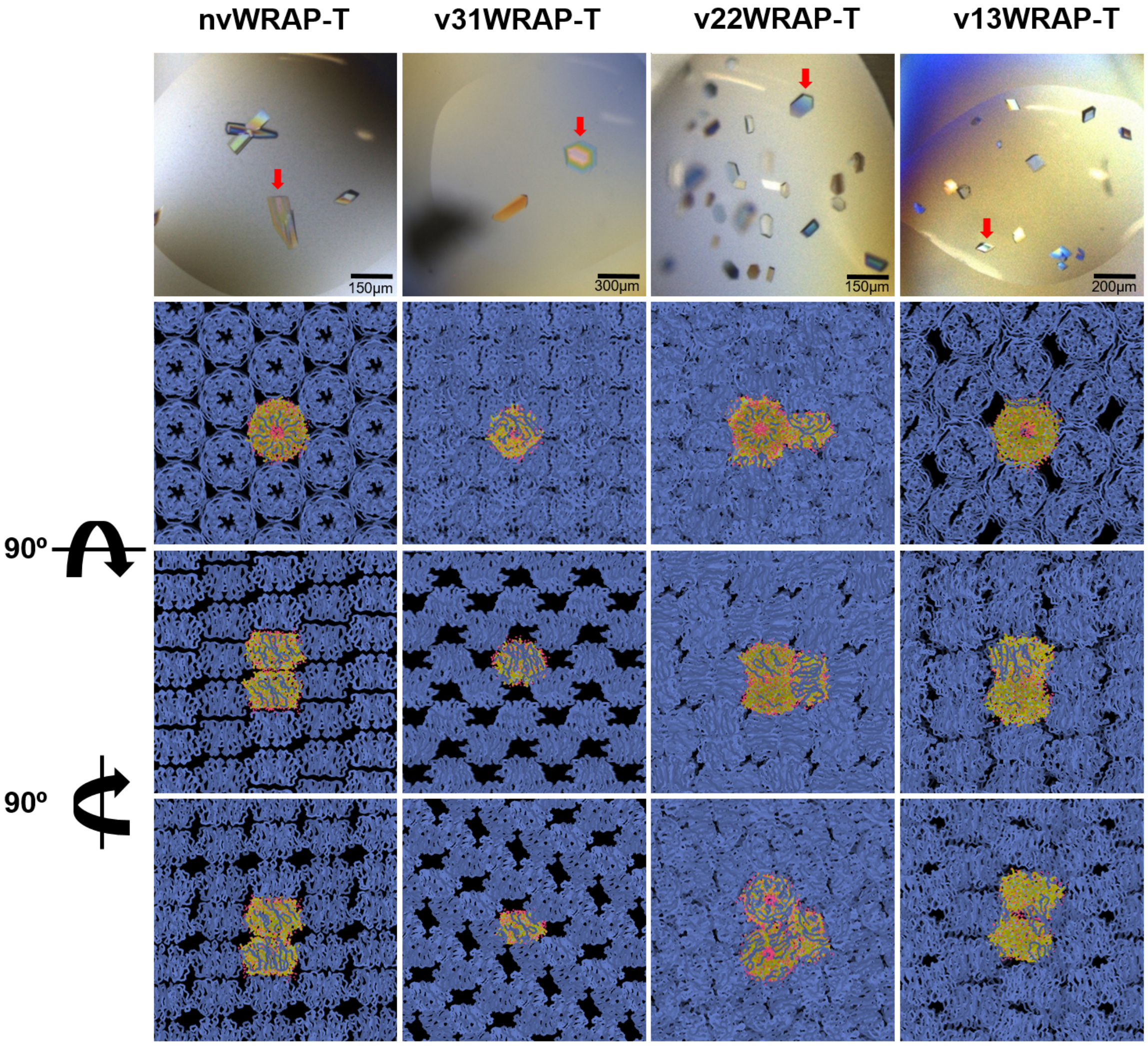

Figure S1: Pictures of all the diffracted crystals and their corresponding crystal packings viewed from the three main axes. The crystal packing was visualised with the COOT software.

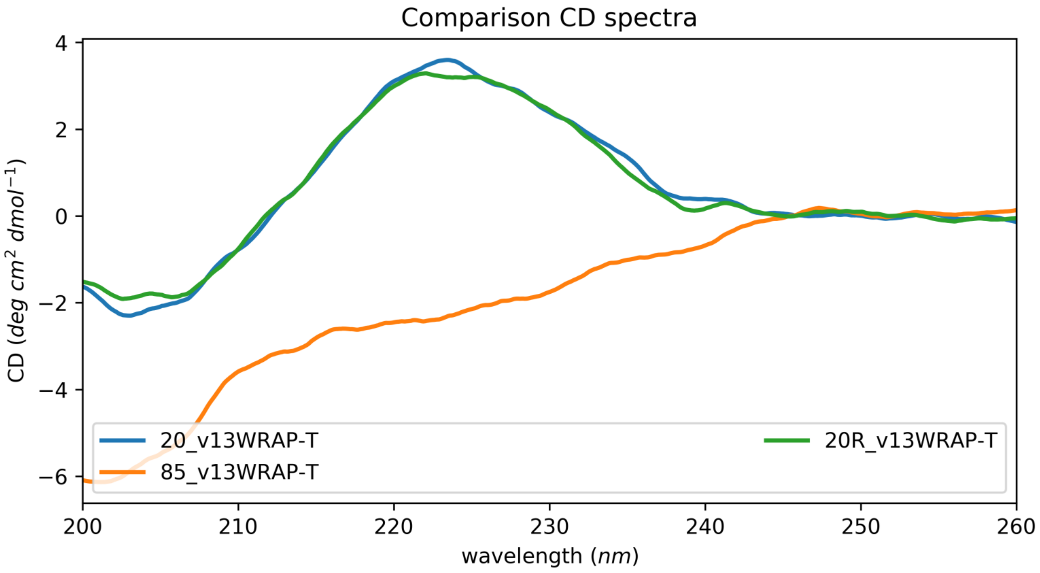

Figure S2: CD spectra of v13WRAP-T at twenty degrees Celsius (blue) and after heating till 85 degrees Celsius (orange). The latter clearly shows the unfolding of the protein. After cooling down to 20 degrees Celsius (green), the protein refolds, showing this process is reversible.

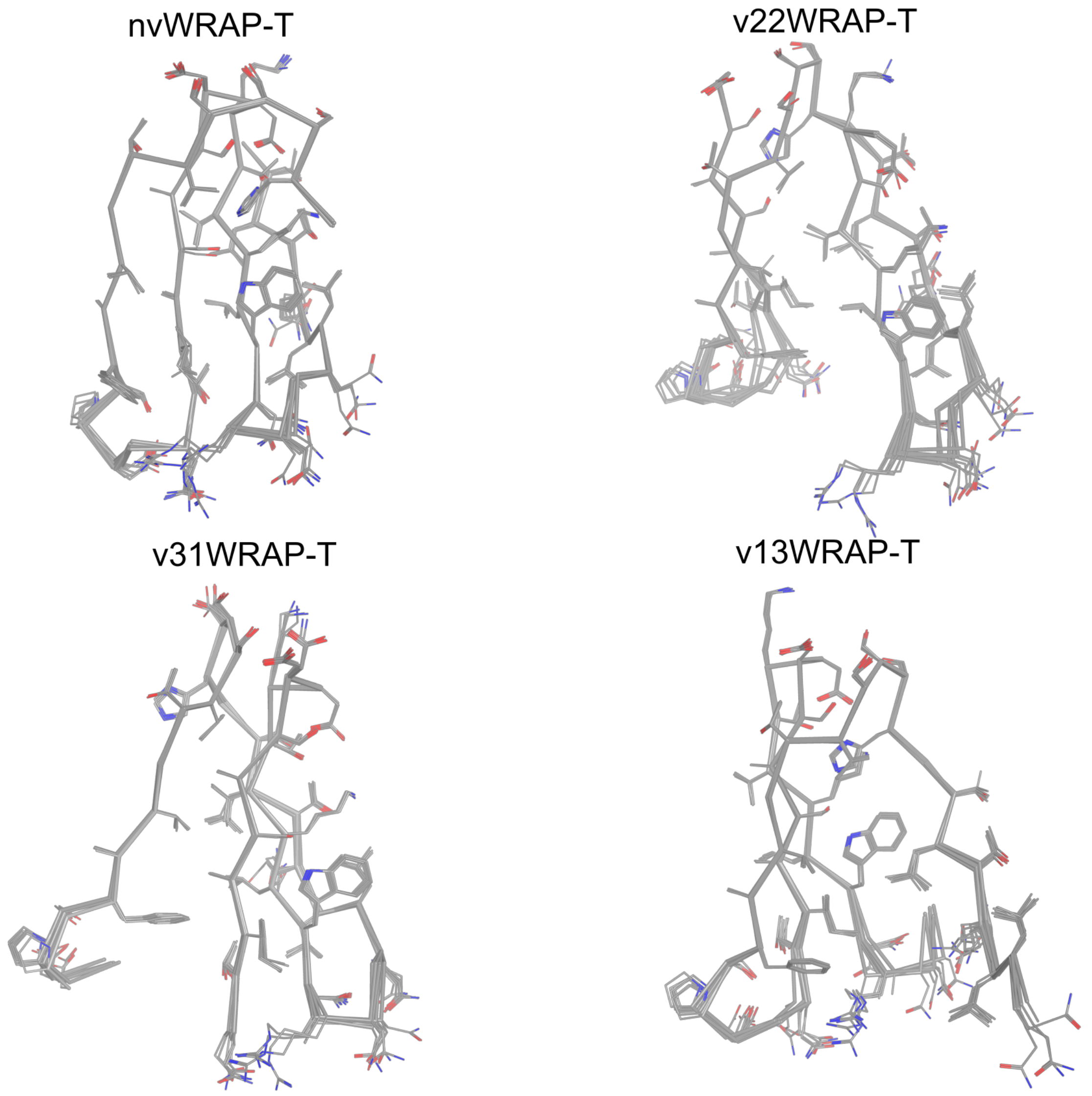

Figure S3: Alignment of all blades of each protein with side chains shown. These illustrate the high symmetry that exists between individual blades as only the outside flexible side chains show major deviations. These figures were created with PyMOL.

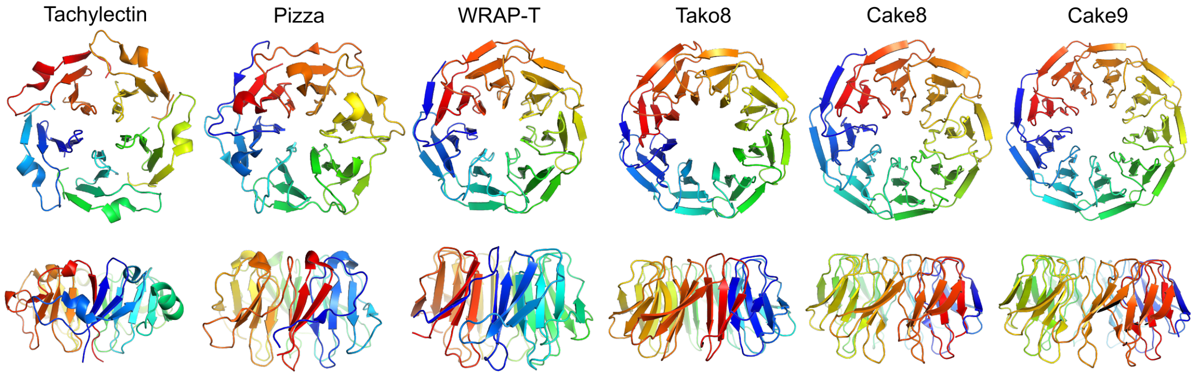

Figure S4: Top and side view of all proteins used in the geometrical comparison: the five-fold Tachylectin-2 (PDB:5c2m), the six-fold Pizza (PDB:3ww9), seven-fold WRAP-T (PDB:7big), eight-fold Tako (PDB:6g6n) and Cake (PDB:6tjg) and the nine-fold Cake (PDB:6tjh). These Figures were created with PyMOL.

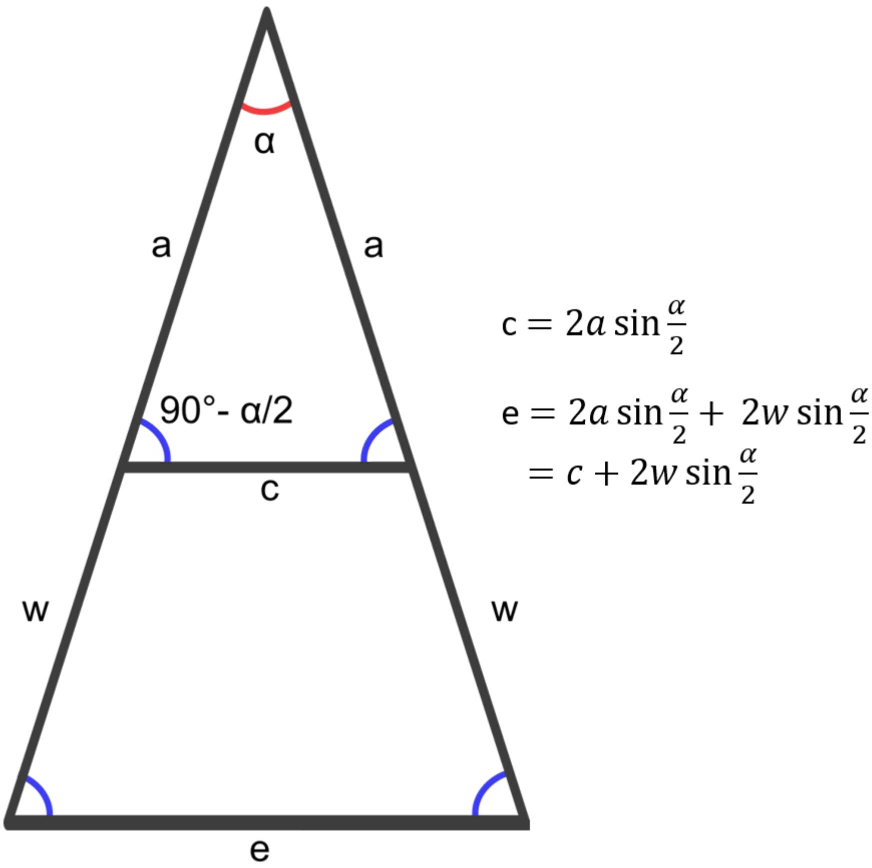

$$c = 2a\sin\frac{\alpha}{2}$$

$$e = 2a\sin\frac{\alpha}{2} + 2w\sin\frac{\alpha}{2}$$
$$= c + 2w\sin\frac{\alpha}{2}$$

Figure S5: Simple model that shows how the interblade distances (c/d and e/f) can be calculated from the channel radius (a/b) and the rotation angle. The w is the distance between the first and third   -strand and remains nearly constant for all propellers.
